# Status quo and influencing factors of job burnout among residents in standardized training

**DOI:** 10.3389/fpubh.2024.1470739

**Published:** 2024-12-16

**Authors:** Jiale An, Yu Chang, Xiyuan Zhang, Minghao Zhang, Xia Lei, MingYi Yang, Yunfeng Hu

**Affiliations:** ^1^Nuclear Industry 215 Hospital of Shaanxi Province/215 Hospital of Shaanxi Province, Xian’yang, Shaanxi, China; ^2^General Family Medicine, The First Affiliated Hospital of Yan’an University, Yan’an, Shaanxi, China; ^3^Department of Radiation Oncology, The First Affiliated Hospital of Yan’an University, Yan’an, Shaanxi, China; ^4^Department of Gynecology, The First Affiliated Hospital of Yan’an University, Yan’an, Shaanxi, China; ^5^Department of Joint Surgery, Honghui Hospital, Xi’an Jiaotong University, Xi’an, Shaanxi, China

**Keywords:** burnout, resident doctor, standardized training trainees, influencing factors, analysis

## Abstract

**Background:**

With the continuous progress and in-depth implementation of the reform of the medical and health care system, alongside the gradual enhancement of the standardized training framework for residents, such training has become a crucial avenue for cultivating high-level clinicians and improving medical quality. However, due to various constraints and limitations in their own capabilities, residents undergoing standardized training are often susceptible to job burnout during this process. Numerous factors contribute to job burnout, which is closely associated with depression and anxiety. To promote effective standardized training and develop high-quality resident personnel, it is essential to investigate the influencing factors of job burnout and implement strategies to mitigate its occurrence.

**Objective:**

Explore the job burnout and its influencing factors among standardized training residents of Affiliated Hospital of Yan ‘an University, and to provide favorable basis for effective prevention of job burnout.

**Methods:**

An online questionnaire survey was conducted among all standardized training residents at the hospital from June 2020 to September 2022. The evaluation utilized a general situation questionnaire, the Job Burnout Inventory - General Survey (MBI-GS), the Self-Rating Anxiety Scale (SAS), and the Self-Rating Depression Scale (SDS).

**Results:**

A total of 660 valid questionnaires were collected, and 315 people had positive reaction for job burnout, and the detection rate was 47.7%. Difference in gender, marital status, whether they held practicing certificates, participated in work before training, used sleep medication, age, grade, sleep state were the influencing factors of different dimensions of job burnout. 175 people were in a state of anxiety (26.5%), while 357 (54.1%) were depressed. Pearson correlation analysis showed that anxiety and depression were highly correlated with job burnout (*p* < 0.01). The results of multi-factor analysis showed that sleep status, whether to take sleep AIDS, anxiety and depression were the factors affecting the dimension of emotional exhaustion (*p* < 0.01). Income, sleep status, anxiety and depression scores were the influencing factors of job burnout dimension (*p* < 0.05). Sleep status and depression score were the factors affecting the dimension of reduced sense of achievement (*p* < 0.05).

**Conclusion:**

Job burnout is prevalent among residents undergoing standardized training, and the factors influencing it are numerous and diverse. It is closely associated with various elements such as age, income, years of training, sleep quality, anxiety, and depression. Therefore, it is recommended to implement appropriate measures to mitigate the occurrence of job burnout and enhance the quality of standardized training for residents. Such measures may include formulating reasonable training policies and theoretical learning plans, as well as providing mental health interventions.

## 1 Introduction

To promote health services development, standardized training for residents in hospital systems has gradually become a key link in training medical professionals. Medical students of various clinical specialties have since realized the ideal of gradually becoming professional residents, which has cultivated many clinicians with noble professional ethics, theoretical knowledge, and clinical skills.

Medical students of various clinical specialties have since realized the ideal of gradually becoming professional residents, which has cultivated many clinicians with noble professional ethics, theoretical knowledge, and clinical skills.

Through these standardized trainings, residents independently undertake the diagnosis and treatment of frequently occurring diseases and skillfully complete their respective departments’ basic operations. The standardized residents training in hospital is booming in china. During this period, standardized resident training standard manuals have formulated the standardized rotation of resident trainees in various clinical departments. Clinical practice should thus equate to public theory, thereby cultivating excellent physicians with professional ethics and abilities. Meanwhile, professional students taking further studies must learn basic clinical research and technical writing, which then leads to greater occupational pressure.

Job burnout is when individuals with long-term work pressure in customer service fields have no effective way of finding relief from pressure-induced burnout. This includes three dimensions of negative psychological reaction, namely emotional exhaustion, depersonalization (or also known as job neglect), and the reduction of personal achievement (1). Earlier studies have shown that medical staff are prone to job burnout (2), which lead to the decline of their overall work performance and may lead to misjudgment and serious clinical errors. Job burnout may also affect residents’ view of their medical career and their career satisfaction, which is closely related to turnover intention (3).

During the instructional phase, residential scholars are required to complete busy and extensive front-line clinical duties in accordance with the rigorous standard of resident doctors, which imposes substantial pressures. Additionally, they are also pressured to keep learning. Studies have pointed out (4) that residents are more prone to job burnout compared to other medical and health workers. During standardized residents training in hospital, burnout affects the residents’ physical and mental health, the results of efforts toward medical and health system reform, the development of health-related practices, and client satisfaction which is ultimately inseparable. This makes it important to explore job burnout and its influencing factors on residents’ standardized training students to provide clues for effective prevention of job burnout. A questionnaire survey was conducted on 660 residential trainees from 2018 to 2021 class in a hospital to understand the burnout situation of residential trainees and explore the influencing factors, providing a valuable reference for improving the quality of standardized training for Chinese residents.

## 2 Objects and methods

### 2.1 Survey objects

This study selected all the standardized training trainees of Affiliated Hospital of Yan ‘an University from batch 2018 to 2021, including medical master’s degree students and social standardized training personnel as the research objects. The online questionnaire was conducted using the questionnaire Star platform and was only distributed to the resident training group. To ensure the effectiveness of the questionnaire, a total of 667 questionnaires were collected. With 7 unqualified questionnaires were eliminated, 660 valid questionnaires were the ultimately selected. Among these, 190 were male and 470 were female. There were 328 licensed doctors and 332 who had not obtained certificates. The questionnaire was completely anonymous, and the purpose and confidentiality of the survey were explained to the participants before the survey.

### 2.2 Survey methods and contents

#### 2.2.1 Basic information

We designed and collected the baseline data of students, including age, gender and marital status; Occupational factors that affect burnout include income, type of students, grade of students, whether they held medical practitioner qualification certificates, and whether they have participated in work before training. Other factors such as sleep and sleeping patterns/circadian rhythms were included as well.

#### 2.2.2 Burnout state

The Maslach Burnout Scale was used herein, which is the MBI-GS revised and translated by Li Chaping et al. (5). The test contains the three dimensions of Emotional Exhaustion (EE), Depersonalization (DP) and low Personal Accomplishment (PA) and has a total of 15 question items. Emotional exhaustion consists of items 1 to 5, which mainly assess the emotional response caused by excessive work stress. The depersonalization dimension is composed of items 6 to 9, which mainly refers to deliberately keeping a distance between work and work objects and presenting an aloof and ignoring attitude toward work objects and the work environment. The low Personal Accomplishment spanned items 10 to 15, which mainly refers to the evaluation of work ability experience and achievement experience. A 7-level scoring method was adopted to explore frequency, and 0 to 6 points were scored according to the frequency of “never” to “every day.”

The dimension of low Personal Accomplishment was the reverse of the scoring principle. There were 25 critical points for emotional exhaustion, 11 points for job neglect, and 16 points for decreased sense of accomplishment. According to the burnout situation of each dimension, job burnout can be divided into either light, medium, or severe job burnout. When only one dimension is positive, it is mild burnout, two dimensions means moderate burnout, and three dimensions means high burnout. In this survey, the Cronbach’s *α* coefficient of the MBI-GS scale was 0.924, and the Cronbach’s α coefficient of the three dimensions of Emotional Exhaustion (EE), Depersonalization (DP) and low Personal Accomplishment (PA) were 0.946, 0.936 and 0.930, respectively, indicating good internal consistency of the scale. The KMO value of validity analysis was 0.940, and the Bartlett’s spherical test was significant (*p* < 0.001), so the scale was suitable for factor analysis.

#### 2.2.3 Anxiety status

Self-Rating Anxiety Scale (SAS) was used to evaluate the anxiety status of residential trainees. The SAS was compiled by ZUNG (6) in 1965, was widely used in clinical practice, and has good reliability and validity. The scale has 20 items, of which items 5, 9, 13, 17 and 19 are reverse scoring questions, hence the scores are added to the total crude score by the scoring method of 1 to 4 and then multiplied by 1.25 to take the integer and then obtaining the standard score for evaluating the subject’s state of anxiety. The cut-off value of SAS standard score is 50 points, and scores 50 to 59 were classified as mild anxiety. Meanwhile, scores 60 and 69 were classified as moderate anxiety, and scores above 69 were classified as severe anxiety.

#### 2.2.4 Depression status

Depression status was assessed using the Self-Rating Depression Scale (SDS) compiled by ZUNG (6) in 1965, with each item scored at levels 1, 2, 3 and 4. 1 (Never or Occasionally), or 2 (Sometimes), or 3 (often), or 4 (Always). 10 items (2, 5, 6, 11, 12, 15, 16, 17, 18, 20) were scored in reverse, and the 10 items were scored in sequence. The Depressive Severity Index is the cumulative score of each item in a set of 80 (the highest total score) with the index ranging from 0.25 to 1.0; the higher the index, the higher the degree of depression. The score index below 0.50 was not considered as depressed, scores 0.50–0.59 were considered as having mild depression, scores 0.60–0.69 were considered as having moderate to high depression, and scores above 0.70 was considered major depression.

### 2.3 Statistical methods

SPSS 26.0 statistical software was used to analyze the collected data. For the Descriptive analysis, frequency, percentage and mean were used to describe the general situation of the research object. Independent sample *t* test, Chi-square test and variance analysis were then used to analyze the differences in the occurrence of job burnout and the scores of various dimensions of job burnout among different population characteristics. Multiple Logistic regression was used to analyze the influencing factors of each dimension of job burnout. A *p* < 0.05 was considered to be statistically significant.

## 3 Results

### 3.1 Basic information of residents in standardized training

A total of 660 questionnaires were collected, of which 190 were male which accounted for 28.8%, while 470 were female which for 71.2%. The majority of residential trainees under the age of 25 which accounts for 49.8% of the total sample. The other basic information is shown in Table 1.

**Table 1:** Sociodemographic and work-related characteristics of respondents.

Sociodemographic characteristics		*N*	%
Age (years)	<26	329	49.8
	26–30	285	43.2
	>30	46	7.0
Gender	Male	190	28.8
	Female	470	71.2
Marital status	Married	91	13.8
	Single	567	85.9
	Divorced/Widowed	2	0.3
Income	<2000	511	77.4
	2000–4,000	72	10.9
	4,000–6,000	60	9.1
	>6,000	17	2.6
Type of trainee	Members of society	140	21.2
	Postgraduate student	520	78.8
Grade of training	First grade	411	62.3
	Second grade	128	19.4
	Grade three	121	18.3
Certificate	Yes	328	49.7
	No	332	50.3
Work experience	Yes	171	25.9
	No	489	74.1
Sleep state	Very dissatisfied	70	10.6
	Dissatisfaction	104	15.8
	Ordinary	307	46.5
	Satisfaction	140	21.2
	Quite satisfied	39	5.9
Takes sleep medication	Yes	40	6.1
	No	620	93.9

### 3.2 The burnout detection rate of residents in standardized training

In this survey, 315 people had positive job burnout results, and the detection rate of job burnout is 47.7%, of which 260 people had mild burnout (39.4%), 49 had moderate burnout (7.4%), and 6 had severe burnout (0.9%). See Table 2 for details.

**Table 2:** Burnout detection rate of standardized training students of third-grade resident doctors.

Job Burnout	*N*	%
No burnout	345	52.3
Low	260	39.4
Moderate	49	7.4
High	6	0.9
A total of	660	100.0

### 3.3 Univariate analysis of basic job burnout of residents in standardized training

The scores of residential training trainees of different genders in the three dimensions of burnout questionnaire were calculated, and the student’s *t* test was conducted to analyze the differences between male and female residential training trainees in the scores of each dimension of burnout. Results showed significant differences between male and female residential training trainees in the scores of decreased sense of achievement (*p* < 0.05). It is suggested that male residential training students have more serious job burnout than female students in the dimension of reduced sense of achievement.

Student’s *t* test was conducted on the scores of different marriages in the three dimensions of the scale, and results showed that there was a statistically significant difference in the scores of the dimension of decreased sense of accomplishment among marriages (*p* < 0.05). Another student’s *t* test was conducted on the scores of the three dimensions of the scale of whether a practicing certificate was obtained before training, and results again showed that the scores of emotional exhaustion and work neglect had statistical significance (*p* < 0.05).

Student’s *t* test was again conducted for the scores of residents’ participation in work before standardized training in the three dimensions of the scale, where results showed that the scores of the dimension of emotional exhaustion had statistical significance (*p* < 0.05). The same student’s *t* test was also conducted for the scores of the three dimensions of the scale of whether or not to take sleep aids, and the results showed that the scores of the dimension of emotional exhaustion had statistical significance (*p* < 0.05).

Variance analysis was conducted on the scores of different ages in the three dimensions of the scale, where the results showed that the scores of emotional exhaustion, work neglect and reduced sense of achievement were statistically significant (*p* < 0.05). The scores of emotional exhaustion and neglect of work tended to decrease with the increase of age, while the loss of sense of accomplishment was more prevalent in physicians who lived in the hospital for a long time. Results of the variance analysis of the scores of physicians with different seniority in the three dimensions of the scale showed that the scores of emotional exhaustion and work neglect had statistical significance (*p* < 0.05). Univariate ANOVA was performed on the scores of different sleep conditions in the three dimensions of the scale, and the results showed (see Table 3) that the score difference of emotional exhaustion, slow work and reduced sense of achievement had statistical significance (*p* < 0.05).

**Table 3:** Parameters associated with burnout syndrome and its dimensions standardized training trainees of residents.

Score	Emotional exhaustion (EE)	Depersonalization (DP)	professional accomplishment (PA)
Gender			
Male	11.93 ± 6.72	6.96 ± 5.71	23.95 ± 8.70
Female	12.26 ± 6.19	7.10 ± 4.96	21.95 ± 8.10
*t*	−0.609	−0.307	2.813
*p*	0.543	0.759	0.005
Marital status			
married	11.21 ± 6.18	6.64 ± 4.95	24.99 ± 7.93
Single	12.32 ± 6.36	7.13 ± 5.22	22.13 ± 8.32
*t*	−1.555	−0.839	3.060
*p*	0.120	0.402	0.002
Certificate			
Yes	12.79 ± 6.49	7.46 ± 5.17	22.80 ± 8.76
No	11.55 ± 6.15	6.67 ± 5.16	22.26 ± 7.86
*t*	2.511	1.975	0.824
*p*	0.012	0.049	0.410
Participate in job burnout analysis before training			
Yes	11.01 ± 5.88	6.40 ± 4.60	23.41 ± 7.93
No	12.57 ± 6.46	7.29 ± 5.35	22.22 ± 8.44
*t*	−2.797	−1.931	1.613
*p*	0.005	0.054	0.107
Uses sleep medication			
Yes	14.756.33	8.95 ± 5.11	20.43 ± 7.66
No	12.00 ± 6.32	6.94 ± 5.16	22.66 ± 8.35
*t*	2.667	2.389	−1.651
*p*	0.008	0.017	0.099
Age differences			
<26	12.14 ± 6.37	6.99 ± 5.23	21.98 ± 8.20
26–30	12.48 ± 6.13	7.44 ± 5.19	22.68 ± 8.48
>30	10.43 ± 7.27	5.24 ± 4.39	25.48 ± 8.91
*F*	2.072	3.666	3.670
*p*	0.127	0.026	0.026
Grade differences			
First grade	11.24 ± 6.11	6.36 ± 5.01	22.72 ± 8.15
Second grade	14.85 ± 6.82	8.75 ± 5.46	22.45 ± 8.63
Grade three	12.50 ± 5.80	7.66 ± 5.00	21.95 ± 8.58
*F*	16.807	11.781	0.406
*p*	<0.001	<0.001	0.667
Sleep state			
Very dissatisfied	18.43 ± 7.84	11.56 ± 6.44	18.80 ± 10.40
Dissatisfaction	14.64 ± 6.35	8.48 ± 5.59	21.88 ± 8.54
Ordinary	11.69 ± 4.93	6.60 ± 4.14	22.10 ± 7.42
Satisfaction	9.32 ± 4.77	5.39 ± 4.44	24.26 ± 7.60
Quite satisfied	8.31 ± 7.93	4.82 ± 5.93	28.08 ± 8.96
*F*	39.586	24.222	10.270
*p*	<0.001	<0.001	<0.001

### 3.4 Correlation analysis of various dimensions of job burnout and anxiety and depression among residential training students

Doctors have to contend with the daily race against time to save lives. The deteriorating doctor-patient relationship and medical market cannot be resolved by itself. Coupled with a series of challenges in the high-intensity and high-load working environment (7, 8). Previous studies have shown that because of the particularity of their profession, when compared with other occupations or people in the same age group, doctors are at higher risk of depression, anxiety, suicide attempts and other mental diseases, and thus often become high-risk groups (9–12). Similarly, a 2015 report from the University of Arizona noted that most graduate and doctoral students reported “above average” stress, citing issues related to schooling as the main source of their stress (13). The relationship between mentors and postgraduates has also been seen to be closely related to the quality of postgraduate education (14). Data shows that strong, supportive, and positive mentoring relationships between postgraduates and their mentors are significantly related to the reduction of anxiety and depression. Residential trainees must shoulder the responsibilities of doctors and complete the tasks of education and teaching. Under the current high standard and high-quality training model, anxiety and depression are common among residential trainees, and their mental health state needs more attention. As the gatekeeper of public health in the future, the mental health of high-level medical talents and the effectiveness of measures to maintain health are closely related to the growth of the medical field, the confidence of future clinical career and the ability to meet challenges (15).

#### 3.4.1 The detection rate of anxiety and depression among residential trainees

The results of the survey herein showed that 175 people had anxiety, accounting for 26.5%, of which 107, 50, and 18 individuals had mild, moderate and severe depression, respectively. The detection rate of depression among residential training students showed that 357 people had depression, accounting for 54.1%. Among these, 207, 133, and 17 individuals had mild, moderate and severe depression, respectively. See Table 4 for details.

**Table 4:** The detection rate of anxiety and depression among residential trainees (*n* %).

	No	Mild	Moderate	Severe
Anxiety state	485 (73.5%)	107 (16.2%)	50 (7.6%)	18 (2.7%)
Depressive state	303 (45.9%)	207 (31.4%)	133 (20.2%)	17 (2.6%)

#### 3.4.2 Correlation analysis of job burnout and anxiety and depression among residents in standardized training

Results show that 87.3% of residential training students with anxiety have job burnout, and that 42.9% of residential training students with job burnout have anxiety. Pearson correlation analysis explored whether there was a correlation between anxiety and job burnout scores and each dimension of residential trainees. Results showed that anxiety was highly correlated with emotional exhaustion, job neglect and reduced sense of achievement (*p <* 0.01) 0.82.9% of residential training students with depression have job burnout, 46.9% of residential training students with job burnout have depression. Results also show that depression is highly correlated with emotional exhaustion, job neglect and reduced sense of achievement (*p <* 0.01). See Table 5 for details.

**Table 5:** Correlation analysis of anxiety, depression and job burnout.

	Emotional exhaustion (EE)	Depersonalization (DP)	Professional accomplishment (PA)
Anxiety	0.547**	0.585**	−0.424**
Depression	0.501**	0.553**	−0.462**

**p < 0.01.

Spearman correlation analysis was used to explore the relationship between various dimensions of job burnout and different degrees of anxiety in residential training students. Results showed a correlation between both factors. To explore the relationship between various dimensions of job burnout and different degrees of depression among residential training students, results show a correlation between the two, as shown in Table 6.

**Table 6:** Correlation analysis of different degree anxiety, depression and job burnout.

	Emotional exhaustion (EE)	Depersonalization (DP)	Professional accomplishment (PA)
Anxiety	0.436**	0.462**	−0.357**
Depression	0.458**	0. 451**	−0.363**

**p< 0.01.

### 3.5 Multi-factor analysis of job burnout

The scores of three dimensions of job burnout were taken as the corresponding variables according to references and professional knowledge, age, gender and marital status. Occupational factors that may affect burnout include income, type of students, grade of students, whether they were medical practitioner qualification certificate holders, and whether they have participated in work before training. Sleep status was also considered herein. Possible factors of anxiety state and depression state were then used as independent variables. Following the inclusion criterion (0.05) and exclusion criterion (0.10), stepwise Logistic multiple linear regression analysis was carried out. Dimension of emotional exhaustion such as sleep status, taking sleep aids, anxiety state and depression state scores were used as influencing factors of emotional exhaustion. Dimensions of job burnout such as income were influencing factors of job burnout score. Dimension of reduced sense of accomplishment such as sleep status and depression score were the influencing factors of reduced sense of accomplishment score. The results are shown in Table 7.

**Table 7:** Multiple logistic regression analysis of each dimension and related factors.

Factor	*β*	SE	Wald *χ*2	*p*	OR	95% CI
Emotional exhaustion						
Sleep status	−2.220	0.655	11.473	0.001	0.109	(0.030, 0.392)
take sleep aids	2.070	0.654	10.022	0.002	7.923	(2.200, 28.539)
Anxiety score	0.060	0.021	8.286	0.004	1.062	(0.004, 1.062)
Depression score	0.097	0.034	8.220	0.004	1.102	(1.031, 1.178)
Score for work neglect						
Income	2.480	1.124	4.872	0.027	11.943	(1.32, 108.022)
Decreased sense of accomplishment score						
Sleep status	−0.817	0.341	5.752	0.016	0.442	(0.227, 0.861)
Depression score	−0.093	0.019	23.408	<0.01	0.911	(0.878, 0.946)

## 4 Discussion

### 4.1 The burnout status of residents in standardized training

In this study, the prevalence of Occupational Burnout is 47.7%, moderate burnout with 7.4, and 0.9% for severe burnout, signifying that the scenario for standardized training students of resident physicians within our facility necessitates significant intervention, which substantially impacts the career training of resident students and the clinical function and research of Chinese advanced medical talent. Colleges and universities, their affiliated hospitals, and corresponding administrative departments must attach great importance to this phenomenon to promote the standardized training and talent cultivation of high-quality resident doctors.

### 4.2 Univariate analysis of job burnout among residents of standardized training with different demographic characteristics

For the relationship between gender and job burnout, various researchers have arrived at differing conclusions (16, 17). This study shows that male residential students are more likely to have a decreased sense of achievement than their female counterparts. For differing marital status, married students are more likely to have a lower sense of achievement; this may be because unmarried workers tend to be less pressured, which may explain their lower levels of burnout. Moreover, they have higher interpersonal skills, ability to participate in problem solving with family members, and adaptability (18). Trainees with practicing certificates are more likely to have burnout in the dimension of emotional exhaustion and work neglect than those without certificate. Students who have not been engaged in occupational activity prior to vocational training or postgraduate study are more inclined to encounter emotional exhaustion, whereas those who were engaged in occupational activity beforehand and acquired substantial work experience can better circumvent burnout. However, residential training doctors who have participated in the workforce under the backlog of work pressure for a long time, experienced setbacks and blows, and have lost their enthusiasm for work and have more obvious emotional exhaustion experience more burnout than their counterparts.

The influence of sleep on work status is also obvious. Sleep was found to affect job burnout, emotional exhaustion, job neglect, and a reduced sense of achievement. Taking sleep medication was seen to aid sleep quality to a certain extent and affected emotional exhaustion and job burnout. The standardized training of resident doctors usually lasts 3 years, and the training is completed after passing the examination. The univariate variance analysis of job burnout in each grade found that residential trainees were prone to emotional exhaustion and neglect of work in the second year. During clinical training, the first year started the learning stage, students first needed to adapt to the environment and rules and regulations of various departments, with less undertaking of clinical tasks. During the final year of the training, students needed to complete a variety of theoretical and skill assessment, graduate students also need to strengthen their scientific research tasks. They could also plan to train second-grade students can devote themselves to clinical career, who are more prone to job burnout.

### 4.3 The influence of anxiety and depression on job burnout

Anxiety is a kind of fear and anxiety in a specific environment, which is a relatively common emotion. Depression, meanwhile, is the expression of negativity, pessimism, loss of interest, among others. Studies have demonstrated that there exists a correlation between depression, anxiety, and other symptoms and job burnout (19), and that there is a covariance rate between burnout and anxiety as well as depression, although the sequence of burnout and depression has not been clearly established. Burnout has also been shown to be associated with depression and problems with clinical performance, and that burnout and depression often occur together; burned-out residents seem to have more doubts about their own abilities and performance. The results of this survey showed that 175 people in the total survey had anxiety, accounting for 26.5%; 357 (54.1%) were depressed. In the correlation analysis, anxiety and depression were significantly correlated with job burnout in all dimensions, indicating the close influence of anxiety and depression on job burnout. In the multivariate analysis, anxiety state was correlated with the three dimensions of job burnout. Depression was only associated with reduced sense of achievement. Depressive symptoms had a negative impact on the process of job burnout.

Given the overlap between burnout and depression, future studies should combine the burnout scale and depression scale to evaluate job burnout. In large comprehensive tertiary hospitals, resident physicians are playing an increasingly significant role by participating in various clinical works and experiments and assuming the role that combines those of clinicians, researchers, and students. This makes them prone to anxiety and depression, which has a considerable correlation with job burnout. As health service providers, their own mental health should thus warrant further scholarly attention.

Along with the negative psychological effects, resident physician job burnout also leads to a lack of interest in work and in professional confidence. Even with irreparable consequences, studies have found that burnout is associated with serious clinical errors. This has a significant impact on the health recovery of patients and on the development and quality of training of resident doctors. Currently, treating burnout is a resource-intensive task in its experimental stage (20). Hence, the best remedy is to prevent the occurrence of job burnout. For relevant departments and hospital management, reasonable training policies and theoretical learning plans can be formulated, and relevant activities can be regularly executed to alleviate the psychological burden of residential trainees and prevent the occurrence of job burnout. For trainees with severe job burnout, appropriate intervention measures can be taken to help them face negative emotions and challenges and delay the further occurrence of job burnout.

Along with providing students with relevant theoretical knowledge and practical opportunities, teachers must also pay more psychological attention to make students grow and mature continuously during training. For trainees themselves, they must understand that they are qualified residents during the training period. Under the careful guidance of the teachers, their knowledge base will be expanded and they will subject under great pressure at the same time. They must thus learn to balance life and work, adjust their emotions and improve their abilities, making it necessary to investigate the influencing factors of the burnout of resident doctors. Based on the hospital and the trainees’ own circumstances and influencing factors, effective preventive intervention measures should be formulated to alleviate the pressure of the working environment, enhance work efficiency, adjust the psychological state of the trainees, and ultimately achieve better standardized training outcomes of resident doctors.

There remains an ongoing discussion as to whether psychosocial stress in residents has a direct or long-term impact on either patients or the doctors themselves, and given the goals of residency training, some stress seems inevitable (21) or even beneficial, although some studies suggest that residents have a high rate of burnout, a severe stress response, and that burnout may be linked to poor mental health and job performance. The understanding of resident burnout can be enhanced by further rigorous studies (22), such as large samples of resident physicians in well-planned prospective studies. The job characteristics that physicians face are complex and vary by specialty, program, and graduate year, and a study aimed at describing burnout must be large enough (or deliberately specific) and prospective to control for these variables and identify risk factors. Future prospective studies may also explore the temporal relationship between the onset of burnout and depression, suicidal ideation, adverse clinical manifestations, substance abuse, career decisions, job dismission, and patient satisfaction.

## Data Availability

The original contributions presented in the study are included in the article/supplementary material, further inquiries can be directed to the corresponding author.
